# Effect of Flavonoids in Hawthorn and Vitamin C Prevents Hypertension in Rats Induced by Heat Exposure

**DOI:** 10.3390/molecules27030866

**Published:** 2022-01-27

**Authors:** Wei Du, Hong-Min Fan, Yu-Xin Zhang, Xiao-Hua Jiang, Yun Li

**Affiliations:** 1 School of Public Health, North China University of Science and Technology, 21 Bohai Road, Caofeidian Xincheng, Tangshan 063210, China; wei_du_vivi@sina.com (W.D.); hongminfan1004@ncst.edu.cn (H.-M.F.); 2 Nutrition and Cardiovascular Diseases Basic Research Group, North China University of Science and Technology, 21 Bohai Road, Caofeidian Xincheng, Tangshan 063210, China; 3 School of Basic Medical Sciences, North China University of Science and Technology, 21 Bohai Road, Caofeidian Xincheng, Tangshan 063210, China; zhangyuxin@ncst.edu.cn (Y.-X.Z.); jiangxiaohua@ncst.edu.cn (X.-H.J.); 4 Key Laboratory for Chronic Diseases, North China University of Science and Technology, 21 Bohai Road, Caofeidian Xincheng, Tangshan 063210, China

**Keywords:** hypertension, oxidative stress, heat exposure, hawthorn flavonoids, vitamin C, physical effort, rats

## Abstract

Background: Excessive oxidative stress is associated with hypertension in professional high-temperature working conditions. Polyphenols exhibit a cardioprotective effect. Hawthorn contains high amounts of flavonoids, though its effect on hypertension protection has yet to be studied. This study aims to investigate this effect of extract of hawthorn (EH) or its combination with vitamin C (Vit. C) in rats induced by working under a hot environment. Methods: Forty-two male rats were randomly divided into a control group under normal temperature and six treatment groups exposed at 33 ± 1 °C along with 1 h of daily treadmill running. They were orally provided with water, Vit. C (14mg/kg), EH (125, 250, and 500 mg/kg), and EH500 + Vit. C, once a day for four weeks. Results: Both EH and Vit. C alone reduced the systolic and diastolic blood pressure of rats exposed to the heat environment; however, their joint supplementation completely maintained their blood pressure to the normal level throughout the experimental period. No morphological changes were found on the intima of aorta. Moreover, the co-supplementation of EH and Vit. C prevented the changes of heat exposure in inducing oxidative stress markers, such as glutathione peroxidase, catalase, total antioxidant capacity, and nitric oxide; the synergistic action was more effective than either individual treatment of EH and Vit. C. Furthermore, the administration of EH had more potent effects on increasing superoxide dismutase, IL-2, the 70 kilodalton heat shock proteins and high sensitivity C reactive protein, and decreasing serum malondialdehyde and lipofuscin in vascular tissue than those in Vit. C group. Conclusions: A strong synergistic effect of EH and Vit. C on the prevention of hypertension under heat exposure was established, as they inhibited the oxidative stress state. This study also sets up a novel intervention strategy in animal models for investigation on the early phases of hypertension induced by heat exposure.

## 1. Introduction

Prevention of hypertension is essential to reduce cardiovascular complications [1]. Excessive reactive oxygen species (ROS) are the main causes of intracellular oxidative stress [2] underlying molecular mechanisms of heart diseases [3,4]. Consumption of a diet rich in antioxidants can decrease the oxidative stress and reduce the risk of hypertension induced by harmful environmental factors, such as heat exposure [5,6,7]. Oxidative stress is caused by the excessive production of oxygen free radicals (O_2_^●−^) and decreasing antioxidant capacity [8,9]. Working or exercising in a hot environment results in heat stress, which has direct impact on cardiac stroke volume and systolic function. Increased cardiac output during whole-body heating is caused by increasing oxygen needs, thermoregulation, dehydration, and hyperthermia. These adjustments result in significant cardiovascular strain [10,11]. Our previous studies found a high prevalence of hypertension among heat-exposed workers who also had higher vitamin C (Vit. C) deficient rates due to its loss via sweat [12,13]. Increasing dietary antioxidants is a good solution to prevent hypertension caused by heat exposure. For example, studies have reported that natural antioxidants, fruits, and vegetables did reduce hypertension through increased nitric oxide bioavailability and diminished inflammatory reactions in the vasculature [14,15,16,17]. Vit. C, a common antioxidant, has been shown to improve blood pressure in animal studies [18,19]. Thus, in this study, Vit. C was used as a positive control reagent.

Studies have shown that phytochemical flavonoids provide a combination of health benefits in the cardiovascular system, such as improving oxidative parameters, inhibiting inflammation, increasing vascular reactivity, and reducing blood pressure [20]. Hawthorn (*Crataegus pinnatifida* Bge.) fruit is widely grown in the north of China, and as homologous in medicine and food used for treatment indigestion for more than 1500 years. In particular, studies in recent years have shown that hawthorn has a stronger antioxidant capacity among drupe fruits due to their oxygen radical absorbance capacity [21]; this positive effect is mainly related to rich flavonoids in hawthorn, which have a large number of hydroxyl groups [22]. Studies have shown that hawthorn, or its leaf extracts, decreases oxidative stress and can be used as a safe, effective, nontoxic agent in the treatment of cardiovascular disease and ischemic heart disease [23,24]. Previous studies have shown green tea extract, lycopene, and Vit. C to have a synergistic antioxidant effect [25], and administration of flavonoids from quercetin, combined with Vit. C had the best effects on improving blood pressure [26]. Studies also indicated that the combination solution of flavonoid extract from *Campnumoea lancifolia* (Roxb.) Merr and Vit. C had excellent cooperative antioxidant activity [27].

The synergistic action of endogenous antioxidants and exogenous natural antioxidants is important in the prevention of disease; it is crucial for the development of intervention strategies for the prevention of cardiovascular diseases induced by hazardous environmental factors shown in this study. Our hypothesis was natural antioxidants of extract of hawthorn inhibit blood pressure induced by heat exposure through the reduction oxidative stress. To verify this hypothesis and evaluate these effects and mechanism, we performed an animal study which simulated the work exposed at high temperature: rats were given, individually or jointly, the equivalent dose conversion of the human upper limit intake of flavonoids from extract of hawthorn and the amount of recommended Vit. C for working in hot environments. Firstly, the antioxidant compounds in the extract of hawthorn was analyzed; secondly the effect of the extract of hawthorn on the blood pressure and morphology of the aortic vascular structure of rats were analyzed; thirdly, the effect of the extract of hawthorn (EH) on the oxidation stress and inflammation biomarkers were measured. The aim of this study is to investigate the antihypertensive effect and its mechanism linked to oxidation stress. This is the first study providing evidence for individual extract of hawthorn and Vit. C, or their combination, in preventing hypertension in workers exposed to high temperatures.

## 2. Results

### 2.1. Antioxidant Compounds in the Extract of Hawthorn

Content of total phenolics, total flavonoids, as well as the main phenolic compounds identification and Vit. C in the extract of hawthorn are listed in Table 1. The antioxidant compounds shown in EH include hyperoside, resveratrol, epicatechin, and proanthocyanidins B_2_.

### 2.2. Effects of Extract of Hawthorn and Vitamin C on Systolic and Diastolic Blood Pressure

**Figure 1:**
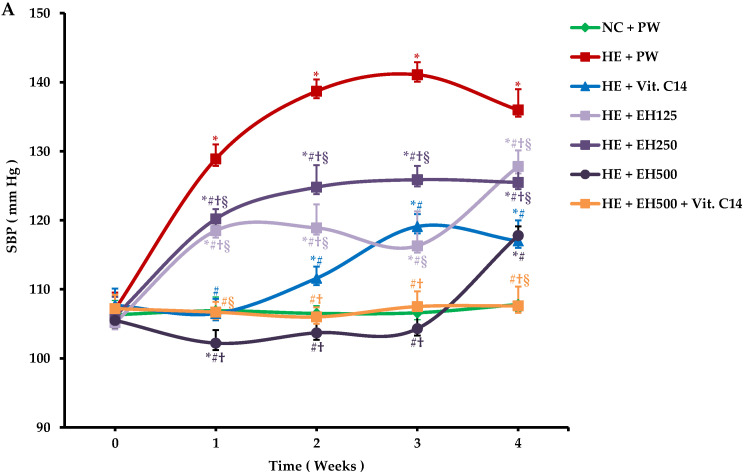

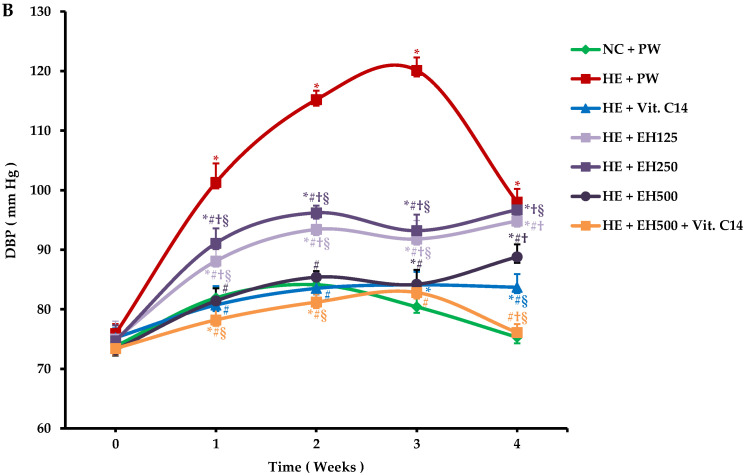
Antihypertensive effect of extract of hawthorn and vitamin C on systolic blood pressure (**A**) and diastolic blood pressure (**B**) during extract of hawthorn and vitamin C administration for 4 weeks. Systolic blood pressure (SBP); diastolic blood pressure (DBP); normal temperature control (NC); heat exposure (HE); purified water (PW); vitamin C (Vit. C); extract of hawthorn (EH). Values are expressed as means ± standard deviation (*n* = 6/group). * indicates *p* < 0.05 vs. NC + PW; # indicates *p* < 0.05 vs. HE + PW; † indicates *p* < 0.05 vs. HE + Vit. C; § indicates *p* < 0.05 vs. HE + EH500.

In the HE + PW group, rats under heat exposure (HE) were given purified water (PW) only, both systolic and diastolic blood pressures had significantly increased tends from day 0 to day 28 compared to the NC + PW group (both *p* < 0.001); here, NC means rats under normal temperature control (Figure 1A,B). On the other hand, compared with the HE + PW group, both systolic and diastolic blood pressure in all HE + EH groups (extract of hawthorn, with dose of 125, 250, and 500 mg/kg, respectively) decreased significantly at day 28 (all *P <* 0.01); moreover, in the HE + EH500 group, they were significantly more reduced than both HE + EH125 and EH250 (all *P <* 0.001). There was no difference between the HE + EH500 group and HE + Vit. C group (HE rats were given Vit. C (14 mg/kg).

### 2.3. Effects of Extract of Hawthorn and Vitamin C on the Wall Thoracic Artery

**Figure 2:**
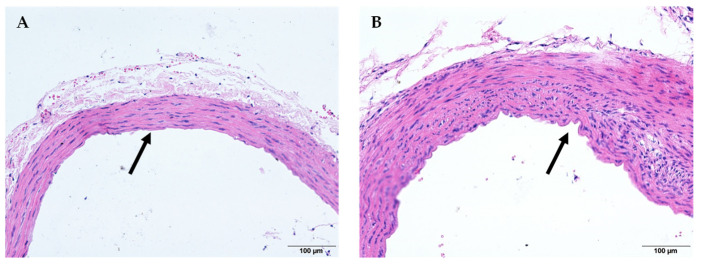

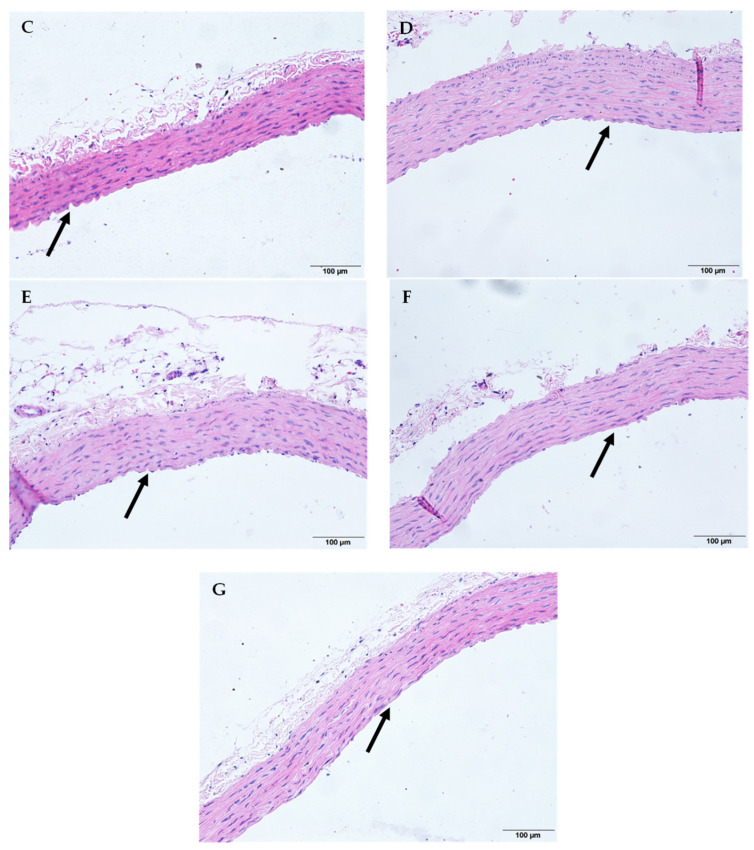
Effect of extract of hawthorn and vitamin C on morphology of aortic vascular structure of rats. NC group (**A**), HE group (**B**), HE + Vit. C group (**C**), HE + EH125 group (**D**), HE + EH250 group (**E**), HE + EH500 group (**F**), and HE + EH500 + Vit. C group (**G**) (H&E × 200). Normal temperature control (NC); heat exposure (HE); purified water (PW); vitamin C (Vit. C); extract of hawthorn (EH).

The vascular morphology of the arterial tissue and the histopathological changes in rats induced by heat exposure are shown in Figure 2. The histopathological morphology in the HE + PW group is shown in Figure 2B, which shows the thickening of the media layer of the artery wall missing smoothing of the endodermis, and the irregular arrangement of muscle cells apparently being seen. However, these were significantly prevented in rats supplemented with EH and/or Vit. C (Figure 2C–G). Interestingly, co-administration of EH of 500 mg/kg and Vit. C of 14 mg/kg shows the strongest effect (Figure 2G), and the single administration of EH of 500 mg/kg (Figure 2F) shows the closest results to the normal morphology of the NC + PW group (Figure 2A). However, the smooth edge of endodermis was lost but irregular cells were still observed in the single Vit. C and both HE + EH125 and HE + EH250 groups (Figure 2C–E).

### 2.4. Effects of Extract of Hawthorn and Vitamin C on Markers of Oxidative Stress

**Figure 3:**
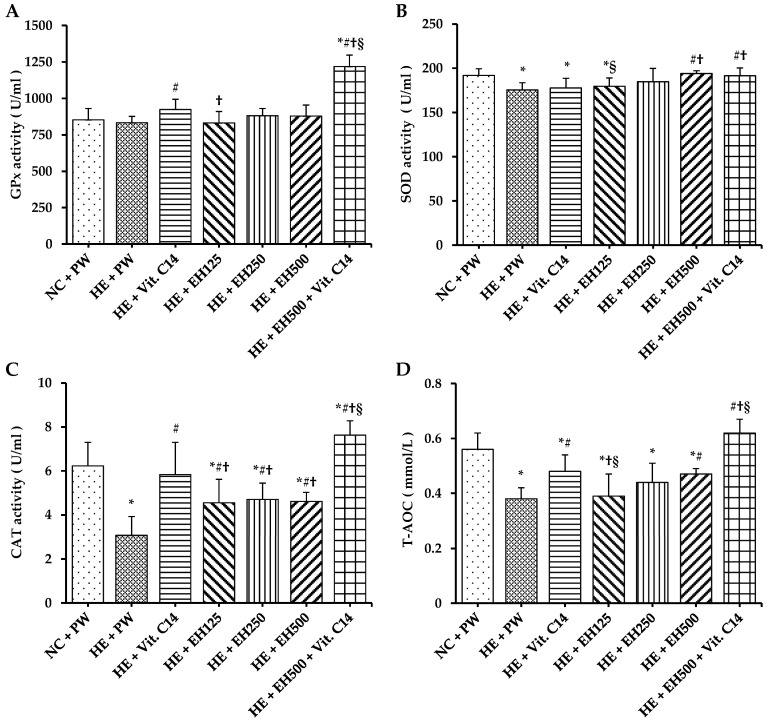
Effects of extract of hawthorn and vitamin C on serum GPx activity (**A**), SOD activity (**B**), catalase activity (**C**), and T-AOC (**D**) in all experimental groups. Glutathione peroxidase (GPx); superoxide dismutase (SOD); catalase (CAT); total antioxidant capacity (T-AOC); normal temperature control (NC); heat exposure (HE); purified water (PW); vitamin C (Vit. C); extract of hawthorn (EH). Values are expressed as means ± standard deviation (*n* = 6/group). * indicates *p* < 0.05 vs. NC + PW; # indicates *p* < 0.05 vs. HE + PW; † indicates *p* < 0.05 vs. HE + Vit. C; § indicates *p* < 0.05 vs. HE + EH500.

**Figure 4:**
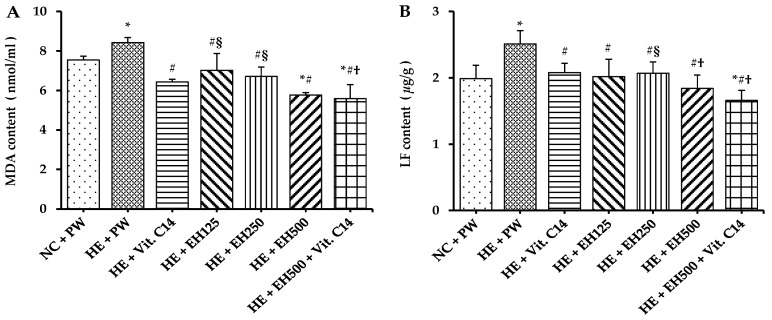
Effects of extract of hawthorn and vitamin C on serum MDA content (**A**), and vascular lipofuscin content (**B**) in all experimental groups. Malondialdehyde (MDA); lipofuscin (LF); normal temperature control (NC); heat exposure (HE); purified water (PW); vitamin C (Vit. C); extract of hawthorn (EH). Values are expressed as means ± standard deviation (*n* = 6/group). * indicates *p* < 0.05 vs. NC + PW; # indicates *p* < 0.05 vs. HE + PW; † indicates *p* < 0.05 vs. HE + Vit. C; § indicates *p* < 0.05 vs. HE + EH500.

In this study, compared with the normal temperature control, the rats running on treadmill under heat exposure significantly decreased the activities of antioxidant enzymes, such as SOD and catalase, and total antioxidant capacity (T-AOC) in serum (Figure 3B–D), whereas the peroxide product, such as malondialdehyde (MDA) in serum and lipofuscin in vascular tissue was significantly increased (Figure 4A,B) (all *p* < 0.05). However, glutathione peroxidase (GPx) activity was not changed significantly by the heat exposure compared to the normal group, but it was elevated significantly only in HE + Vit. C and HE + EH500 + Vit. C groups (*p* < 0.05 and *p* < 0.001, respectively; Figure 3A); Even more, GPx activity in the HE + EH500 + Vit. C group was significantly higher compared to the HE + Vit. C group (*p* < 0.001), suggesting that a combination of Vit. C and EH500 had a higher synergistic antioxidant effect. SOD activities in both HE + EH500 and HE + EH500 + Vit. C were recovered to the normal level of the NC + PW group even though there was no difference between these two groups (Figure 3B). Catalase activity was significantly reduced in the HE+ PW group but it was increased to normal level in all other groups, either supplemented with EH or Vit.C or their combination (all *p* < 0.05; Figure 3C). Among these groups, rats supplemented with EH (500 mg/kg) combined with Vit. C showed the highest results for both GPx (Figure 3A) and catalase (Figure 3C) compared with the NC + PW group and other groups (*p* < 0.05). Furthermore, T-AOC, the overall antioxidant levels, in the HE group as well as HE groups supplemented with EH 500 mg/kg or Vit. C alone were significantly lower than that in the NC + PW group and HE + EH500 + Vit. C group (all *p* < 0.01; Figure 3D). The groups supplemented with EH/or and Vit. C showed lower levels of serum MDA (Figure 4A) and vascular lipofuscin (Figure 4B) than those in the HE + PW group (all *p* < 0.001); moreover, the group supplemented with HE + EH500 + Vit. C showed lower values of these markers than those in the NC + PW group (both *p* < 0.01).

### 2.5. Effects of Extract of Hawthorn and Vitamin C on the Nitric Oxide Generation

**Figure 5:**
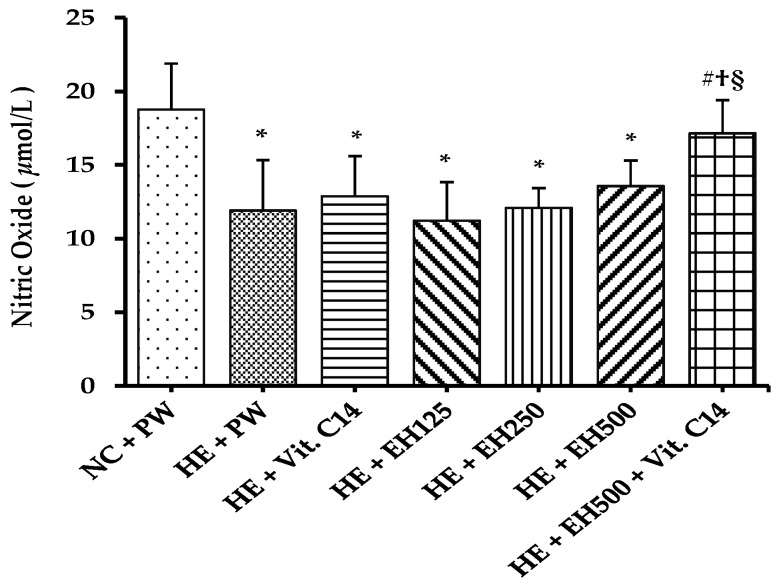
Effect of extract of hawthorn and vitamin C on serum nitric oxide level in all experimental groups. Nitric oxide (NO); normal temperature control (NC); heat exposure (HE); purified water (PW); vitamin C (Vit. C); extract of hawthorn (EH). Values are expressed as means ± standard deviation (n = 6/group). * indicates *p* < 0.05 vs. NC + PW; # indicates *p* < 0.05 vs. HE + PW; † indicates *p* < 0.05 vs. HE + Vit. C; § indicates *p* < 0.05 vs. HE + EH500.

In this study, there was a significant reduction in serum nitric oxide levels in the HE + PW group compared with the NC + PW group (*p* < 0.01; Figure 5) while supplementation only combined with EH 500 mg/kg and Vit. C significantly increased (*p* < 0.05) to the normal level.

### 2.6. Effects of Extract of Hawthorn and Vitamin C on the Inflammatory and Protective Factors

**Figure 6:**
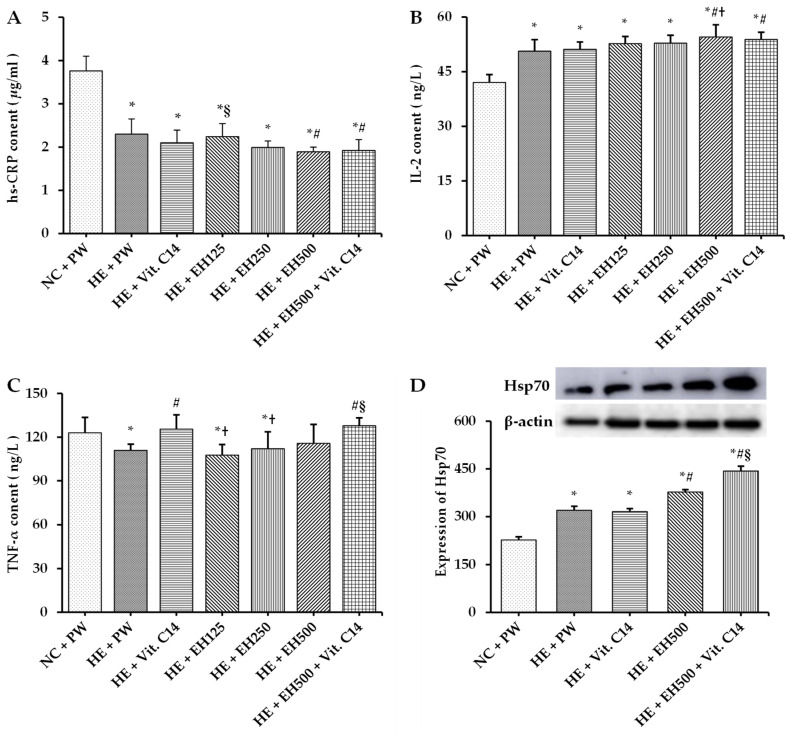
Effects of extract of hawthorn and vitamin C on serum hs-CRP (**A**), IL-2 (**B**), TNF-α (**C**) levels, and tissue-specific expression of Hsp70 (**D**) in all experimental groups. High sensitivity C reactive protein (hs-CRP); interleukin 2 (IL-2); tumor necrosis factor-α (TNF-α); heat shock protein 70 (Hsp70); normal temperature control (NC); heat exposure (HE); purified water (PW); vitamin C (Vit. C); extract of hawthorn (EH). Values are expressed as means ± standard deviation (*n* = 6/group). * indicates *p* < 0.05 vs. NC + PW; # indicates *p* < 0.05 vs. HE + PW; † indicates *p* < 0.05 vs. HE + Vit. C; § indicates *p* < 0.05 vs. HE + EH500.

Interestingly, serum hs-CRP level was significantly reduced in all HE groups with or without EH or/and Vit. C supplementation compared with the NC group (all *p* < 0.001; Figure 6A), suggesting that hs-CRP, as a proinflammatory parameter, did not contribute to arterial wall damage in rats exposed to heat (Figure 2B). Furthermore, the level of serum IL-2 and the expression of heat shock protein 70 (Hsp70) (can protect cells from thermal or oxidative stress) in liver tissue were significantly increased in all HE groups with or without EH or/and Vit. C supplementation compared with the NC group (all *p* < 0.001; Figure 6B,D). However, supplementations with EH (500 mg/kg) alone or combined with Vit. C significantly decreased the level of serum hs-CRP in heat-exposed rats (both *p* < 0.05; Figure 6A). Moreover, these supplementations increased the level of IL-2 and expression of Hsp70 (all *p* < 0.05; Figure 6B), but these effects were not obvious in the Vit. C group, indicating that the anti-inflammatory effect of IL-2 and Hsp70 was due to the effect of EH. However, serum TNF-α level was significantly decreased in the HE + PW group compared with the NC + PW group (*p* < 0.05; Figure 6C); this level was significantly increased in both groups with Vit. C supplementation individually or combined with EH (both *p* < 0.05), indicating the anti-inflammatory effect of TNF-α is caused mainly by Vit. C action.

## 3. Discussion

Heat exposure leads to excessive oxidative stress, inducing vascular endothelial remodeling and increased blood pressure, as previously reported [28], as well as excessive loss of antioxidant nutrients in sweat because of body heat regulation as we have previously found [13]. Many studies have shown the benefits of antioxidant supplementary interventions, which can elevate antioxidant capacity and reduce the lipid oxidation, thus reducing vascular damage to avoid severe cardiovascular consequences, such as hypertension. Our supplementary intervention study indicates that EH alone or combined with Vit. C can elevate antioxidant capacity and mitigate lipid oxidation, reduce vascular pressure and avoid severe cardiovascular consequences, such as an improvement in systolic and diastolic blood pressure along with normalization in the arterial wall in running rats exposed to heat stress.

The supplementation with extract of hawthorn (500 mg/kg) as well as Vit. C, individually or in combination, suppressed the increased blood pressure in heat-exposed rats. These protective effects may be mainly associated with the uses of antioxidants, such as Vit. C [29] and flavonoids in extracts [30]. Interestingly, co-administration of EH (500 mg/kg) and Vit. C (14 mg/kg) can efficiently maintain the blood pressure level to a nearly normal control status throughout the experimental period along with the normalization in the morphology of arterial wall to alleviate vasoconstriction, as shown in Figure 2C–F; these changes clearly indicate that EH plays a major role in improvement of vascular remodeling after heat stress. Their ability to maintain the intact arterial wall would contribute to the antihypertension effect [3,4]. For the first time, a combination supplementation of Vit. C and extract of hawthorn completely prevented heat exposure-induced blood pressure elevation. The mechanism of this effect was further analyzed by the antioxidant capacity of EH or the combination of EH and Vit. C in the following analysis.

Anti-oxidation ability has been shown to be reduced when the body is exposed to a hot environment [12,13]. Indeed, our results clearly indicated that rats running on a treadmill under heat exposure significantly decreased their activity in SOD, catalase, and T-AOC, whereas they showed increases in the peroxide products, MDA, and lipofuscin, which led to decreased oxidation resistance and increased lipid peroxidation. These may contribute to arterial wall damage and elevated blood pressure in this condition. The GPx activity was elevated significantly only in HE + Vit. C and HE + EH500 + Vit. C groups, especially in the HE + EH500 + Vit. C group, suggesting that the combination of Vit. C and EH500 had a higher synergistic antioxidant effect. This synergistic effect also showed in the changes the activities of SOD and catalase, as well as T-AOC, the overall antioxidant levels. Hawthorn flavonoids increased the activities of antioxidant enzymes, which may be related to the regulation of antioxidant enzyme expression by flavonoids. Antioxidant enzymes may be associated with the endogenous antioxidant stress pathway-nuclear factor-E2-related factor 2 (Nrf2)/antioxidant response element pathway, which regulates the expression of genes encoding downstream antioxidant enzymes. For example, epicatechin can induce the activation of the Nrf2 signaling pathway, and Nrf2 enters the nucleus and binds to the antioxidant response element to enhance the expression of antioxidant proteins [31], while supplementation with EH/or and Vit. C showed lower levels of serum MDA and vascular lipofuscin, especially in the group supplemented with HE + EH500 + Vit. C. The changes of these antioxidant parameters plus the improvement of vascular remodeling suggests that co-administration of these antioxidants completely prevented the damage caused by excessive ROS.

Vitamin C is a key antioxidant and acts as a strong antioxidant to protect against oxidative damage by donating its two hydrogens to free radicals. High quality polyphenols exist in hawthorn (Table 1), and they contain large amounts of hydroxyl groups related to its strong oxygen radical absorbance capacity [21]. These may be associated with the stronger antioxidant activity as previously reported [32]. EH may, through polyphenols, possess an antioxidant effect to protect vascular and anti-inflammatory activities, which is consistent with a previous report [23], to reduce the risk of CVDs, including hypertension [33]. Even more, a combination of various nutrients will counteract oxidative stress-induced cardiovascular risk factors [19]. In this study, the partial depression of systolic and diastolic blood pressure was shown and complete inhibition of the vascular wall impairment as a consequence of supplementation with EH was found to be more significant than those of Vit. C in heat-exposed rats. Vit. C and flavonoids are ideal natural antioxidants that participate in the synergy action of antioxidant networks or multi-antioxidant chains [34]. In this study, the synergistic action of Vit. C and EH maintained the activities of SOD, GPx and catalase in rats exposed to hot environment. The present preventive results are consistent with the previous study of Ajibade et al. [26], who reported quercetin and Vit. C mitigated cobalt chloride-induced hypertension through a reduction in oxidative stress in rat and verified the synergistic antioxidant effect of Vit. C combined with plant flavonoid extract [25].

Endothelial dysfunction is an early stage in the development of hypertension [16]. Nitric oxide is produced by cells and works as a vasodilator to relax the inner muscles of blood vessels. The decreasing of nitric oxide indicates the reduction in nitric oxide synthesis and bioavailability which is associated with many cardiovascular risk factors, such as hypertension [35]. There is association between impairment of endothelial function and nitric oxide levels reductions. In this study, the group of supplements combined with EH 500 mg/kg and Vit. C significantly increased serum nitric oxide. These data indicated that coadministration of EH and Vit. C increased nitric oxide bioavailability and, as a consequence, prevented vascular damage in rats exposed to the hot environment.

In other studies, chronic heat exposure-induced endothelial dysfunction was associated with decreased nitric oxide production and marked with medial wall thickening [36], smooth vascular endodermis missing and lost tightly arranged muscle cells [37], ultimately leading to a significant increase in blood pressure [38,39]. Nitric oxide works as a messenger of endothelial cells in regulating vascular tension and vascular remodeling in hypertensive rat models [14,40]. ROS can uncouple endothelial nitric oxide synthase (eNOS) and produce O_2_^●−^ rather than nitric oxide, which decreases the nitric oxide level by the endothelium [14]. In this study, the level of nitric oxide increased and normalized arterial wall were only found in the rats administered both Vit. C and EH group, this further suggests the protective roles by the combination of Vit. C and EH dietary antioxidants, at least in part is through reducing the nitric oxide levels for the normalized blood pressure observed in the heat-exposed rats.

The inflammatory reactions caused by oxidative stress can induce vascular injury accompanied with high hs-CRP [41,42]. In this study, we did not find that inflammation was a manifestation after heat exposure. Serum hs-CRP level was significantly reduced in all HE groups with or without EH or/and Vit. C supplementation, this suggests that hs-CRP, as a proinflammatory parameter, did not contribute to arterial wall damage in rats exposed to heat, at least in this case. But the level of IL-2 in serum and Hsp70 in liver tissue were significantly increased in all HE groups with or without EH or/and Vit. C supplementation. These suggested that these anti-inflammatory factor and protective cytokines may contribute to the decrease of hs-CRP in these heat-exposed rats. Serum TNF-α level was significantly decreased in HE + PW group while increased in both groups with Vit. C supplementation individually or combined with EH, indicating the anti-inflammatory effect of TNF-α is caused mainly by Vit. C action. Although the serum hs-CRP level was not increased after heat exposure, its level was significantly reduced after EH supplementation, such a protective effect may be partly due to the upregulation of protective cytokines such as Hsp 70 expression, and anti-inflammatory cytokines such as IL-2 levels. These may also contribute to the normalization of the arterial wall as Vit. C and EH confer beneficial effects on cardiovascular health [43,44].

In the present study, the level of inflammatory biomarker hs-CRP in serum was decreased significantly in all heat-exposed rats provided with or not provided with EH or Vit.C, which was inconsistent with the media layer of the artery wall impairment in these rats. A plausible interpretation can be that the modest increases in other protective cytokines and anti-inflammatory cytokines following heat-induced oxidative stress, are responsible for the relative decrease in proinflammatory cytokine CRP levels [45], such as increased Hsp70 expression [46], and increased IL-2 levels may be regulated by lower levels of messenger nitric oxide in rats undergoing heat stress. This result is in line with some previous studies in animal models, which showed a negative correlation between the anti-inflammatory effects of flavonoids and serum hs-CRP. These flavonoids include quercetin, grape seed proanthocyanidins, and anthocyanins [47,48,49]. Accordingly, increased serum IL-2 and upregulation of liver Hsp70 expression in rats supplemented with EH 500 mg/kg alone or with Vit. C were found. There was an increase in TNF-α in rats supplemented with Vit. C, which may also partly be contributed to the normalization of the arterial wall in heat-exposed rats consumed with EH and Vit. C. Previous studies have shown that the beneficial effect of fresh orange juice rich in Vit. C intake decreases hs-CRP in healthy volunteers [50]. The above results suggest that EH can protect against endothelial damage and has a protective effect on controlling inflammatory reactions [51].

A close association has been described for arteriolar tone and the level of plasma H_2_O_2_ in hypertensive rats [52]. Catalase is a fundamental enzyme in maintaining cellular H_2_O_2_ at appropriate levels. Either extract of hawthorn or vitamin C clearly elevated the catalase level, which agreed with earlier reports by Zheng et al. [53] and Vasdev et al. [54]. Moreover, the synergistic effects of the antioxidant and extract contributed to more catalase activity exceeding the control, which is consistent with the increased nitric oxide of this group. This positive result assists in scavenging H_2_O_2_ and controlling the level of O_2_^●−^ available to react with nitric oxide, which contributes to a net effect of vasodilatation [55,56]. It is probable that Vit. C and flavonoids rich in hawthorn extract induced vasodilation and consequently lower blood pressure by counteracting this pathway. The observations in this study were consistent with previous reports. For instance, hawthorn extract has been reported to protect against high salt-induced hypertension in Dahl salt-sensitive rats by impacting oxidative stress and metabolic patterns [53]. Likewise, prophylactic ingestion of vitamin C effectively abrogated peripheral vascular dysfunction following exposure to 60% O_2_ in an experiment in humans [57]. Taken together, the data indicate significant antioxidant properties of extract of hawthorn combined with vitamin C, conferring endothelial protection in heat-exposed rats and preventing hypertension.

## 4. Materials and Methods

### 4.1. Animals

Male Sprague Dawley rats (200 to 220 g) were obtained from Huakang Bioscience Co., Ltd. (Beijing, China). The animals were kept in polypropylene boxes (340 × 200 × 410 mm) under a humidity and temperature-controlled animal breeding cabinet. Three rats were in the same cage. The animal study was carried out in the Laboratory Animal Centre, North China University of Science and Technology, Tangshan city, China. All animals were maintained under standard environmental conditions with controlled temperature (20–25 °C), light/dark cycles of 12 h, and relative humidity of 50–60%. Rats accessed water and a standard diet (Chang Sheng Co., Ltd., Benxi, China) ad libitum. All procedures were performed according to the ethical principles and institutional guidelines, as well as the International Guide for the Use of Animals in Biomedical Research.

### 4.2. Ethical Considerations

The study was conducted according to the guidelines of the Declaration of Helsinki. The project was referred to the Ethics Committee on Animal Use of North China University of Science and Technology (protocol code LX2018139).

### 4.3. Vitamin C and Extract of Hawthorn

Vitamin C was obtained from Aladdin Co., Ltd. (Shanghai, China; l(+)-ascorbic acid; 99% pure; crystal) and was of analytical grade. EH was purchased from Yishengxiang Co., Ltd. (Lanzhou, China). The EH was a fine powder product with pale yellow–brown color. It was kept at 4 °C to avoid light during the entire experimental period. The components of antioxidants were analyzed by the following methods. Briefly, the samples were treated with 70% ethanol by ultrasonic cleaning, then they were filtered on 0.45 mm nylon membranes. Total phenolics content was analyzed by Folin–Ciocalteu reagent, and total flavonoids content was analyzed by using 5% aluminium nitrate and 5% sodium hydroxide and assessed by spectrophotometry (Shimadzu UV-1780, Tokyo, Japan) at 778 nm and 510 nm. Phenolic and flavonoid concentrations were estimated by correlating the absorbance of the samples to a standard curve made with gallic acid and rutin, and the results were expressed as mg gallic acid equivalent (GAE)/g extract and mg rutin equivalent (RE)/g extract. The content of phenolic compound in the extract was analyzed by high-performance liquid chromatography (Waters UPLC I-Class TQ-S, Milford, MA, USA), using ACQUITY UPLC^®^HSS T3 column 250 mm × 4.6 mm and 5.0 µm for analysis of the resveratrol and proanthocyanidins B_2_ at 306 nm and 210 nm, respectively, and using the column of 100 mm × 2.1 mm and 1.8 µm for analysis of the hyperoside, epicatechin, chlorogenic acid, and ferulic acid at 280 nm, according to their elution order and by comparing their retention time with the respective standard. The results were expressed as mg/g extract. The Vit. C content in the extract was determined by 2,6-dichlorindophenol titration.

The doses administered to rats were obtained from the animal equivalent dose (AED) conversion based on the ratio of animal and human specific surface area and reference intakes for humans, such as AED (mg/kg) = Human does (mg/kg) × K_m_ ratio [58], K_m_ is specific surface area (SSA), SSA (m^2^/kg) = body surface area (BSA)/weight. Here, rat BSA (m^2^) = K × W^2/3^, W is weight (kg) while K is a conversion coefficient, which is 0.09 for rat; human BSA (m^2^) = 0.0061 × H + 0.0128 × W − 0.1529, H is height (cm), W is weight (kg). As the aim of the study was to investigate the effect of flavonoids in hawthorn for human workers in a high temperature environment, and according to our previous results from a nutrition survey of male steel workers [13], we used the reference for human BSA as height to be 175.3 cm and weight to be 78.5 kg. Thus, for a rat with weight of 0.2 kg, its K_m_ ratio is 6.12. In this study, the supplementation of Vit. C (14 mg/kg) in rats was based on the upper limit of the recommended Vit. C intake of 180 mg/d of a worker working in a hot environment [59]. Flavonoid supplementation of 62.37 mg/kg in rats was based on a tolerable upper intake level of proanthocyanidins of 800 mg/d in Chinese DRI (2013) [60], which was equivalent to a EH supplementation of 495 mg/kg in rats, according to 125.9 mg/g total flavonoids compounds determined in the extract. Thus, we set the high dose of EH as 500 mg/kg.

### 4.4. Experimental Conditions

For the simulation of working at high temperature, a heat-exposed treadmill for the rats was established, and a treadmill under normal temperature was used as control. In this experiment, heat exposure was a treatment factor, and treadmill running was a nontreatment factor in all groups of rats. The running platform (ZH-PT, Zhenghua Biological Instrument Equipment Co., Ltd., Huaibei, China) was set with a tilt angle of 15°, a rotational speed of 14 m/min, according to Bedford’s animal treadmill training experiment theory [61], and the treadmill motion model [62]. A wet bulb globe temperature (WBGT) index measurement instrument (3M Quest QT-36, Sao Paulo, MN, USA) was used to monitor the temperature around the treadmill. Normal temperature was adjusted at WBGT 22 ± 1 °C by an air conditioner. High temperature was maintained at 33 ± 1 °C by two halogen tubes (850 W–1199 W), approximately 1 m away from the treadmill. The temperature was simulated based on the WBGT index of a steel worker exposed to high temperature in his working environment [13]. The rats were placed on the platform, running for 0.5 h each in the morning and in the afternoon, daily. After the running, rats were returned to their cabinet.

### 4.5. Experimental Procedures

After 1 week of acclimatization, rats were randomly divided into one normal temperature control (NC) group and six heat exposure (HE) groups, each group had six rats. Group 1: NC group, rats were given purified water (PW); Group 2: HE group, HE rats were administered with only PW; Group 3: HE + Vit. C, HE rats were administered with Vit. C (14 mg/kg); Group 4–6: HE + EH, HE rats were administered with EH 125 mg/kg, 250 mg/kg, and 500 mg/kg respectively; Group 7: HE + EH500 + Vit. C, HE rats were administered with a combination of both EH (500 mg/kg) and Vit. C (14 mg/kg). Administrations were performed once with a gavage needle (Gauge, 16, YA0192, Solarbio Life Sciences Co., Beijing, China) in the morning for 28 consecutive days, and the maximum volume given was 1 mL of solution for each 100 g of body weight. The solutions were prepared daily, and ultra-pure water was used as a vehicle or the control at the same volume. After administration, rats received a fixed 1 h (0.5 h each in the morning and in the afternoon) treadmill running in a normal temperature environment (for NC group) or a heat-exposed environment (for HE groups) with daily treadmill running, except on the day of blood pressure measurement. Blood pressure recordings were performed at baseline and at the end of the week. Rats were sacrificed and samples were collected on the 29th day of the commencement of the experiment.

### 4.6. Blood Pressure Measurement

Systolic blood pressure and diastolic blood pressure were measured indirectly in conscious rats by tail plethysmography using a rat noninvasive blood pressure meter (BP-2010A; Softron Biotechnology Co., Ltd., Beijing, China). Three readings with less than a 5 mm Hg difference were used to calculate the average, which was considered a valid value for a rat in the quiescent state following acclimatization.

### 4.7. Samples Collection

The rats were anaesthetized with 10% chloral hydrate (0.03 mL/kg, intraperitoneal). Subsequently, blood samples and tissues were rapidly collected for assays of antioxidant markers, Western blot, and histopathology. The thoracic aorta and liver tissue were removed, rinsed in saline, and immediately kept on ice. The rats were decapitated using a guillotine. The blood was collected, and the serum was separated. Sera and liver tissues were kept at −80 °C until further analysis.

### 4.8. Vascular Tissue Preparation

Vascular tissue samples were homogenized in ice-cold normal saline (10 volumes for the vasculature) with a Teflon homogenizer. The homogenates were centrifuged at 12,000× *g* for 5 min in a centrifuge (5804R, Eppendorf, Hamburg, Germany) at 4 °C. The supernatant was removed and stored at −80 °C for analysis. The thoracic aorta tissues were collected in 10% neutral formalin saline buffer for proper fixation for 24 h. These tissues were processed and embedded in paraffin wax. Sections of 5–6 μm in thickness were made and stained with haematoxylin and eosin (H&E) for histopathological examination under an optical microscope (SteREO Discovery V20, Jena, Germany) [63].

### 4.9. Biochemical Analyses

#### 4.9.1. Assay of Lipid Oxidation

Lipid oxidation was evaluated by the end production of MDA in serum and lipofuscin in vascular tissues, which indirectly reflects the severity of free radical attack on the cells [34,35]. MDA level was measured by thibabituric acid (TBA) reactive substances following a previously described method [4], and determined by an assay kit (Jiancheng Bio., Nanjing, China); a colored product was detected spectrophotometrically at 532 nm (UV-1780 spectrophotometer; Shimadzu, Japan), and expressed as nmol/mL. The concentration of lipofuscin in vascular tissue was determined by an enzyme-linked immunosorbent assay (ELISA) kit (Jianglai Bio., Shanghai, China), and absorbance was measured in a microplate reader (VERSAmax, San Francisco, CA, USA) at 450 nm. The results were expressed as μg/g vascular tissue.

#### 4.9.2. Assay of Antioxidant Enzyme Activity

Antioxidant enzyme activity indirectly represents the ability to scavenge oxygen free radicals, which is inversely correlated with oxidative damage to the body, as previously described [34]. The activity levels were determined by an assay kit (Jiancheng Bio., Nanjing, China), and the results of activity were expressed as U/mL serum. Determination of superoxide dismutase (SOD) activity was based on the inhibition of the reaction of hydroxylamine with the superoxide radical anion produced through the reaction systems of xanthine and xanthine oxidase. A standard curve with known concentrations of SOD (5000 U/mg; Boehringer Mannheim, Mannheim, Germany) was used. SOD activity can be determined by measuring the rate of oxidized nitrite formation, which leads to the formation of a purplish red product detected with a spectrophotometer at 550 nm. The 50% inhibition rate of SOD in 1 mL reaction solution was expressed as 1 U activity. Determination of glutathione peroxidase (GPx) activity was based on the promotion of the reaction of reduced glutathione (GSH) with hydrogen peroxide (H_2_O_2_). GPx activity was measured through the evaluation of GSH consumption, and the reaction of the remaining GSH with dithiodnitrobenzoic acid led to the formation of a yellow product detected with spectrophotometer at 412 nm. A 1 mol/L concentration of GSH reduced 0.1 mL serum and was expressed as 1 U GPx activity, excluding nonenzymatic reactions. Catalase activity was measured through the evaluation of H_2_O_2_ consumption, and the reaction of the remaining H_2_O_2_ with ammonium molybdate led to the formation of a light yellow complex compound detected with a spectrophotometer at 405 nm. H_2_O_2_ (1 µmol) reduced 0.1 mL serum per second and was expressed as 1 U catalase activity. T-AOC is the expression of overall antioxidant levels of macromolecules, small molecules, and enzymes in the antioxidant system [64]. T-AOC in samples was measured using the ferric reducing/antioxidant power (FRAP) assay. Antioxidant substances in acidic solution return ferric-tripyridyl-triazine (Fe^3+^-TPTZ) to Fe^2+^-TPTZ of the blue product detected spectrophotometrically at 593 nm. A standard curve of 100 mM FeSO_4_-7H_2_O was used. The results were expressed as mmol/L serum.

#### 4.9.3. Assay of Serum Nitric Oxide

Nitric oxide in serum was measured using the nitrate reductase reaction principle [14]. The absorbance of the final staining was detected at 550 nm by a spectrophotometer. The concentration of nitrite in the sample was determined by an assay kit (Jiancheng Bio., Nanjing, China) from a standard curve with 100 μmol/L sodium nitrite (NaNO_2_) solution prepared freshly, and was expressed as μmol NO_2_^−^/L serum.

#### 4.9.4. Assay of Serum hs-CRP, IL-2, and TNF-α

The serum hs-CRP, interleukin 2 (IL-2), and tumor necrosis factor-α (TNF-α) were quantified using a solid-phase sandwich ELISA kits (Jiancheng Bio., Nanjing, China) following the supplier’s instructions as previously described [65]. The absorbance of the samples was read at 450 nm on a microplate reader (Winooski, VT, USA). The hs-CRP level was expressed as μg/mL serum, the IL-2 and TNF-α level were expressed as ng/L serum.

#### 4.9.5. Western Blot Analysis of Hsp70

The 70 kilodalton heat shock proteins (Hsp70) was measured in aortic tissue homogenates following previously described methods with some modifications [56]. The aortic tissue was minced and placed in a lysis buffer, and homogenized by the ultrasonic homogenizer (Bio-Rad Co., Berkeley, CA, USA) on ice-water for 30 s and centrifuged by high-speed freezing centrifuge (5804R, Eppendorf Co., Hamburg, Germany) at 12,000× *g*/min for 5 min at 4 °C to remove insoluble debris. Protein concentrations were analyzed using the BCA reagent (Beyotime Biotechnology, Shanghai, China) in a microplate reader (VERSAmax, San Francisco, CA, USA) at 562 nm. The tissue samples were separated by electrophoresis (JY200C, BIO-RAD Co., Berkeley, CA, USA) in a 10% sodium dodecyl sulfate-polyacrylamide gel. The protein bands were transferred onto a polyvinylidene fluoride membrane. The membrane was blocked with 5% skim milk in tris-buffered saline with 0.1% Tween-20 for 2 h and incubated overnight at 4 °C with primary antibodies: mouse polyclonal anti-Hsp70 (Becton Dickinson Co., San Diego, CA, USA), β-actin, and goat polyclonal immunoglobulin G (Cell Signaling Technology, Danvers, MA, USA). After washing, membranes were incubated with horseradish peroxidise-conjugated secondary antibody for 2 h at room temperature. The membranes were washed and incubated with an enhanced chemiluminescence substrate. The densities of protein of specific bands were visualized with an electrochemiluminescence substrate using a gel imaging system with the image analysis software (Heraeus Co., Hanau, Germany), the value of densities was used as the amount of protein expression.

### 4.10. Statistical Analysis

All analyses were performed using SPSS software version 23.0. The data of the measured variables were normalized by the test, expressed as means ± standard deviation. The difference among groups was analyzed by one-way analysis of variance and complemented with LSD post-test. Repeated measures analysis of variance was used to compare the systolic and diastolic blood pressure measurements for intra-group. Statistical significance was defined as *p* < 0.05.

## 5. Conclusions

In summary, this study tested the antihypertension effect of hawthorn by providing extract of hawthorn to rats, along with daily treadmill running in a 33 °C environment, to mimic a high temperature environment of a worker, e.g., a steel worker’s environment. The extract of hawthorn (500 mg/kg), alone or combined with vitamin C, was able to prevent oxidative damage by reducing the oxidative stress markers; their synergistic action completely prevented the damage to the endothelium of aorta and maintained normal systolic and diastolic blood pressure. The mechanism of these effects was attributed to their strong antioxidant capacity by the enhancement of the antioxidant enzymes’ activities and nitric oxide generation as well as the elimination of peroxide production to maintain the redox balance and prevent vascular damage. Moreover, extract of hawthorn can also reduce inflammation. This study provides the first evidence in a rat model that regular consumption of extract of hawthorn for rats working under heat exposure can prevent hypertension through compromising redox homeostasis. The synergistic protective action is even stronger if rats consumed both extract of hawthorn and vitamin C. Even more, this study sets up a novel intervention strategy in a rat model for investigation into the early phases of hypertension induced by heat exposure. This model can contribute extensively to a better understanding of oxidative stress under heat exposure.

## Figures and Tables

**Table 1:** Main antioxidant compounds in the extract of hawthorn.

Antioxidant Ingredients	Content
Total phenolic compounds (mg GAE/g extract)	264.7
Total flavonoids compounds (mg RE/g extract)	125.9
Phenolic compounds identification (mg/g extract)	
Hyperoside	3.240
Resveratrol	0.149
Epicatechin	0.130
Chlorogenic acid	0.115
Proanthocyanidins B_2_	0.053
Ferulic acid	0.006
Vitamin C (mg/g extract)	0.760

Note: GAE, gallic acid equivalent; RE, rutin equivalent.

## Data Availability

All data generated or analyzed during this study are included in this article.

## Sample Availability

Samples of the compounds are available from the authors.
